# The *CUC1* and *CUC2* genes promote carpel margin meristem formation during *Arabidopsis* gynoecium development

**DOI:** 10.3389/fpls.2014.00165

**Published:** 2014-04-30

**Authors:** Yuri Kamiuchi, Kayo Yamamoto, Masahiko Furutani, Masao Tasaka, Mitsuhiro Aida

**Affiliations:** Department of Plant Biology, Graduate School of Biological Sciences, Nara Institute of Science and TechnologyIkoma, Japan

**Keywords:** *Arabidopsis thaliana*, carpel margin meristem, shoot meristem, leaf development, MicroRNA (miRNA), fruit development

## Abstract

Carpel margin meristems (CMMs), a pair of meristematic tissues present along the margins of two fused carpel primordia of *Arabidopsis thaliana*, are essential for the formation of ovules and the septum, two major internal structures of the gynoecium. Although a number of regulatory factors involved in shoot meristem activity are known to be required for the formation of these gynoecial structures, their direct roles in CMM development have yet to be addressed. Here we show that the *CUP-SHAPED COTYLEDON* genes *CUC1* and *CUC2*, which are essential for shoot meristem initiation, are also required for formation and stable positioning of the CMMs. Early in CMM formation, *CUC1* and *CUC2* are also required for expression of the *SHOOT MERISTEMLESS* gene, a central regulator for stem cell maintenance in the shoot meristem. Moreover, plants carrying miR164-resistant forms of *CUC1* and *CUC2* resulted in extra CMM activity with altered positioning. Our results thus demonstrate that the two regulatory proteins controlling shoot meristem activity also play critical roles in elaboration of the female reproductive organ through the control of meristematic activity.

## Introduction

In the plant shoot, leaves and floral organs are produced from the shoot and floral meristems, respectively. These meristems maintain pluripotent stem cells at the center and differentiate appropriate types of lateral organs at their periphery depending on the developmental context. Although diverse in their shape and function, floral organs (sepals, petals, stamens, and carpels) are considered to be modified leaves and their specific characters are conferred by combinatorial actions of homeotic genes (Krizek and Fletcher, 2005). How unique shapes of individual organ types are generated is a central question in understanding plant shoot development.

The carpel is a component of the gynoecium, a highly complex organ system dedicated to reproduction. Either single or multiple carpel(s) fuse to form the gynoecium and enclose ovules inside. Ovules are formed by meristematic tissues located within or adjacent to carpel primordia (Yamaki et al., 2011) and in *Arabidopsis thaliana*, they are produced by a pair of meristematic tissues called carpel margin meristems (CMMs; also called medial ridges), which are present along the fused margins of the two carpel primordia (Nole-Wilson et al., 2010). In addition to producing ovules laterally, the CMM pair terminates and fuses with each other along their tip, forming the septum that acts as transmitting tissue for pollen tubes.

Several regulators of shoot meristem activity are involved in carpel margin development (reviewed in Reyes-Olalde et al., 2013). The *REPLUMLESS* gene (*RPL*; also known as *BLR* and *PNY*) encoding a BELL-type homeodomain protein is expressed in carpel margins and is required for replum development (Roeder et al., 2003). The RPL protein physically interacts with a class I KNOX protein BREVIPEDICELLUS (BP) and the activity of the two proteins counteracts with *JAGGED* (*JAG*), *FILAMENTOUS FLOWER* (*FIL*), *YABBY3* (*YAB3*), and *ASYMMETRIC LEAVES1/2* (*AS1* and *AS2*) genes, which promote the fate of adjacent valve and valve margin tissues (Dinneny et al., 2005; Alonso-Cantabrana et al., 2007; Gonzalez-Reig et al., 2012). Both *RPL* and *BP* genes are also expressed in the shoot meristem and affect internode length (Smith and Hake, 2003). The RPL protein interacts with another class I KNOX protein SHOOT MERISTEMLESS (STM) and they act together to maintain stem cells in the shoot meristem (Byrne et al., 2003). Although strong mutant alleles of *stm* do not produce flowers due to their strong shoot meristem defects, function of *STM* in carpel development has been accessed using weak *stm* alleles or inducible RNAi plants, which produce abnormal flowers. In these flowers, carpels often fail to fuse at their margins and develop few ovules, indicating that *STM* is required for proper formation of carpel margins (Endrizzi et al., 1996; Scofield et al., 2007).

**Figure 1:**
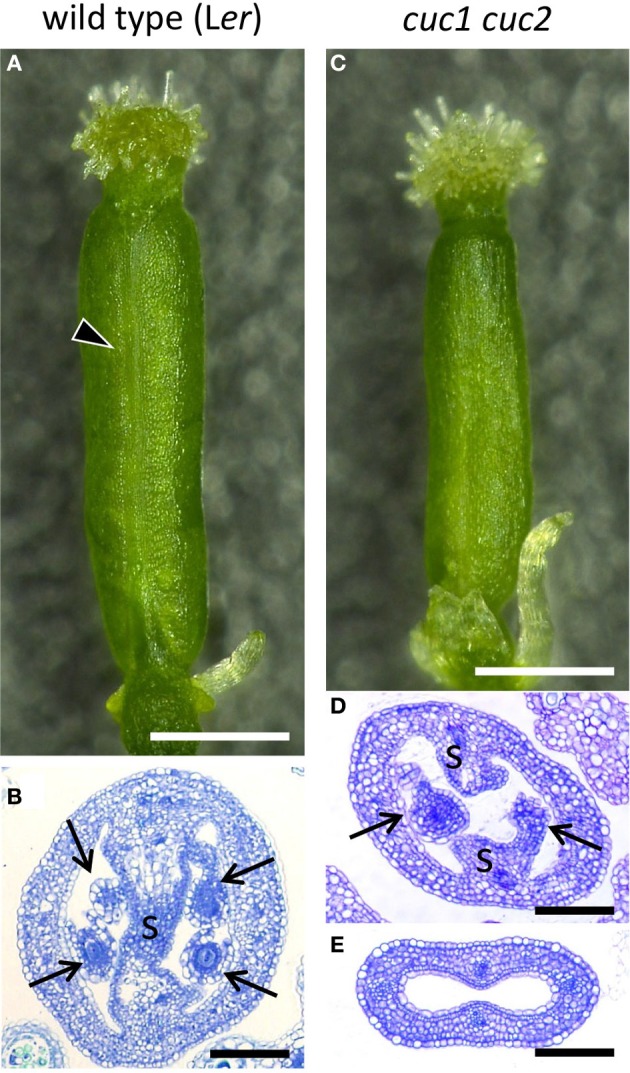
**Gynoecium phenotype of *cuc1 cuc2***. Mature gynoecia from regenerated plants of wild type L*er* **(A,B)** and *cuc1 cuc2* **(C–E)**. Whole view **(A,C)** and transverse sections **(B,D,E)**. Arrowhead in **(A)** indicates the replum. Arrow, ovule; s, septum. Scale bars are: **(A,C)**, 500 μm; **(B**,**D,E)**, 100 μm.

The *CUP-SHAPED COTYLEDON* genes *CUC1* and *CUC2* encoding a pair of paralogous NAC transcription factors are required for shoot meristem initiation through promoting *STM* expression (Hibara et al., 2003). Because of their functional redundancy, seedlings of each single mutant show little morphological phenotype while their double mutants completely lack a shoot meristem and produce severely fused cotyledons. Viable shoots with flowers can be regenerated from double mutant calli and these flowers produce carpels with severe reduction of ovules, septum and replum (Ishida et al., 2000; Figure 1). The Auxin Response Factor MONOPTEROS (MP) is required for *CUC1* and *CUC2* expression possibly through its direct binding to the gene promoters and the *CUC* genes in turn affect expression and polarity of the auxin transport protein PIN1 in ovule primordia (Galbiati et al., 2013). In addition, both *CUC1* and *CUC2* are negatively regulated by the microRNA miR164, which is encoded by three loci in *Arabidopsis*. Disruption of miR164 encoding genes or that of its target sequences in *CUC1* and *CUC2* causes misregulation of their expression, resulting in various developmental defects including abnormal carpel development (Mallory et al., 2004; Baker et al., 2005; Nikovics et al., 2006; Sieber et al., 2007; Larue et al., 2009).

The above results point to the importance of *CUC1* and *CUC2* and in formation of carpel margin structures. However, the developmental basis of the roles for these factors has not been fully investigated. Notably, whether the factors directly affect CMM formation and if so, how they interact during the process remains unknown. Here we investigated the roles for *CUC1* and *CUC2* in gynoecium development by loss and gain of function approaches. The results demonstrate that the *CUC1* and *CUC2* genes are critical for normal CMM development.

## Materials and methods

### Plant materials and growth conditions

The *Arabidopsis thaliana* accessions Landsberg *erecta* (L*er*) and Columbia (Col) were used as the wild type strains. The *cuc1 cuc2* double mutant (*cuc1-1 cuc2-1*) is in the L*er* background (Aida et al., 1997). *CUC2g-m4* is in the Col background and was described previously (Nikovics et al., 2006). For construction of *CUC1g-m7*, the miR164 target sequence (AG CAC GTG TCC TGT TTC TCC A) of *CUC1* was replaced by a mutant sequence (AG CAC GTG AGT TGT TTT AGT A), which contains seven silent mutations (underlined). The mutated genomic fragment corresponding to the nucleotides 5108201..5112019 of chromosome 3 (TAIR 10) was cloned into pGreenII 0229 (Hellens et al., 2000) and transformed into Col. A transgenic line displaying extra petal number and reduced sepal growth, a typical phenotype described for miR164 resistant *5mCUC1* (Mallory et al., 2004), was selected and subjected to analysis. This line accumulated *CUC1* mRNA ~9.5 fold of the wild-type level in inflorescence apices. Seeds were surface sterilized and sown on MS plates as previously described (Fukaki et al., 1996). After incubation for 2 days at 4°C in the dark, plants were grown in a growth chamber at 23°C under constant white light. Ten- to fourteen-day-old seedlings were transferred onto soil and grown at 23°C under constant white light. Induction of calli from root explants and subsequent shoot regeneration was performed as previously described (Aida et al., 1997). Flower stages were determined as previously described (Smyth et al., 1990). Stage 9 was further subdivided into early, mid and late substages, each corresponding to stages 5, 6, and 7 of anther development (Sanders et al., 1999).

### Histological analysis

Histological sections (3 μm) were prepared as previously described(Aida et al., 1997), except that formalin/acetic acid/alcohol (FAA) was used as a fixative. Scanning electron microscopy was carried out as described previously (Aida et al., 1997). *In situ* hybridization was performed as previously described (Ishida et al., 2000) with following modifications: 6 instead of 8 μm sections were prepared and hybridized at 45°C instead of 42°C. Probes for *STM* and *FIL* have been described previously (Long et al., 1996; Sawa et al., 1999). Templates for *CUC1* and *CUC2* probes were the full-length coding sequences. In the wild type and *cuc1 cuc2*, coloring reaction was performed for 36 h, with the initial 12 h at room temperature and the remaining at 4°C. In *CUC1g-m7* and *CUC2g-m4*, coloring reaction was carried out for 12 h at room temperature.

## Results

### *CUC1* and *CUC2* are required for the initiation of the CMMs

**Figure 2:**
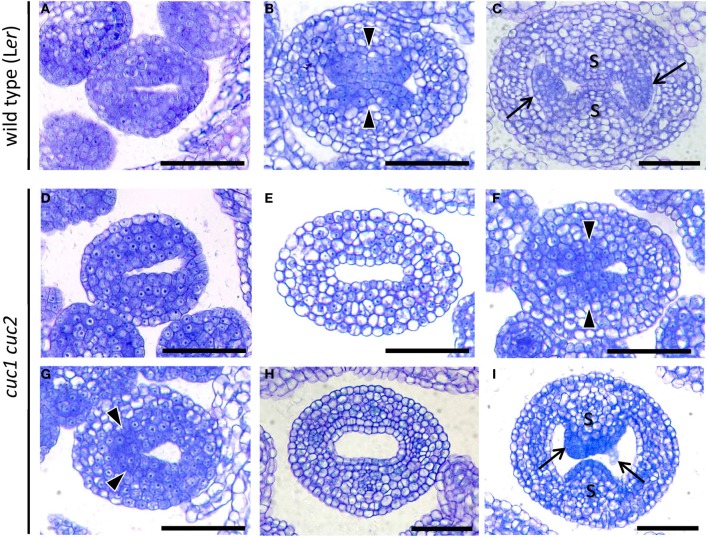
***CUC1* and *CUC2* are required for formation and stable positioning of CMM**. Transverse sections of developing gynoecium at stage 7 **(A,D)**, stage 8 **(G)**, early stage 9 **(B,E,F)**, mid stage 9 **(H)**, and late stage 9 **(C,I)**. Sections are prepared from wild type **(A–C)** and *cuc1 cuc2* **(D–I)**. Arrow, ovule; arrowhead, CMM; s, septum. Scale bars are 50 μm.

To compare gynoecium development of the wild type (L*er*) and *cuc1 cuc2*, we used inflorescence shoots regenerated from calli of each genotype (Aida et al., 1997; Ishida et al., 2000). Wild-type gynoecia in regenerated plants showed essentially the same morphology as those from non-regenerated plants (Figures 1A,B) and followed normal developmental stages (Figure 2; Smyth et al., 1990). Mature gynoecia of *cuc1 cuc2* were somewhat smaller than those of wild type and tended to lose replum tissues most prominently in the apical region of the ovary (compare Figures 1A **and** 1C; Ishida et al., 2000). In histological sections, the septum and ovules were severely reduced (Figure 1D) or completely missing (Figure 1E).

Gynoecium primordia of *cuc1 cuc2* were indistinguishable from those of the wild type up to stage 7, at which both wild type and *cuc1 cuc2* formed a cylindrical primordium consisting mostly of densely cytoplasmic cells (Figures 2A,D). Deviation of the mutant phenotype began at stage 8 to early stage 9, when the wild type initiated two bulges of CMM from the adaxial wall. In the wild-type, cells in the CMMs remained cytoplasmically dense whereas the rest of the cells started vacuolation, which was a sign of cell differentiation (Figure 2B). On the other hand, *cuc1 cuc2* frequently failed to form either one or both of the CMMs and cells in the corresponding regions became vacuolated (Figure 2E; Table 1). In some mutant gynoecia, CMMs were formed on both sides, but their size was smaller than that of the wild type (Figure 2F). In addition, the positioning of CMM initiation was often asymmetric (Figure 2G). When the wild type initiated ovule primordia, cells at the contacting surfaces of the two CMMs underwent post-genital fusion to form the septum (Figure 2C). On the other hand, a significant fraction of *cuc1 cuc2* gynoecia still failed to initiate CMMs (Figure 2H; Table 1). Some mutant gynoecia developed small bumps at the corresponding positions, but their surfaces often failed to contact (Figure 2I; Table 1). These bumps were likely to retain organogenic activity as indicated by the presence of densely cytoplasmic cells, and might produce ovule-like primordia from their flanks. Taken together, these results show that *CUC1* and *CUC2* are required for CMM initiation. Occasional CMM formation at asymmetric positions indicates that the *CUC* gene activities are also required for stable positioning of the CMMs.

**Table 1:** **Effect of *cuc1 cuc2* on CMM formation**.

**Genotype**	Stage^*^	**CMM formation**			CMM contact^**^	
		**Both sides**	**One side**	**None**	**Yes**	**No**
wild type	8	9 (100%)	0	0	8 (89%)	1 (11%)
	Early 9	14 (100%)	0	0	14 (100%)	0
	Mid 9	7 (100%)	0	0	7 (100%)	0
*cuc1 cuc2*	8	3 (33%)	3 (33%)	3 (33%)	3 (100%)	0
	Early 9	7 (39%)	7 (39%)	4 (22%)	7 (100%)	0
	Mid 9	6 (55%)	4 (36%)	1 (9%)	3 (50%)	3 (50%)

* Flower stage (Smyth et al., 1990). Stages early 9 and mid 9 correspond to anther stage 5 and 6, respectively, (Sanders et al., 1999).

** Scored only when CMM ridges are formed on the both sides.

### Expression of *CUC1* and *CUC2* predicts the sites of CMM initiation

**Figure 3:**
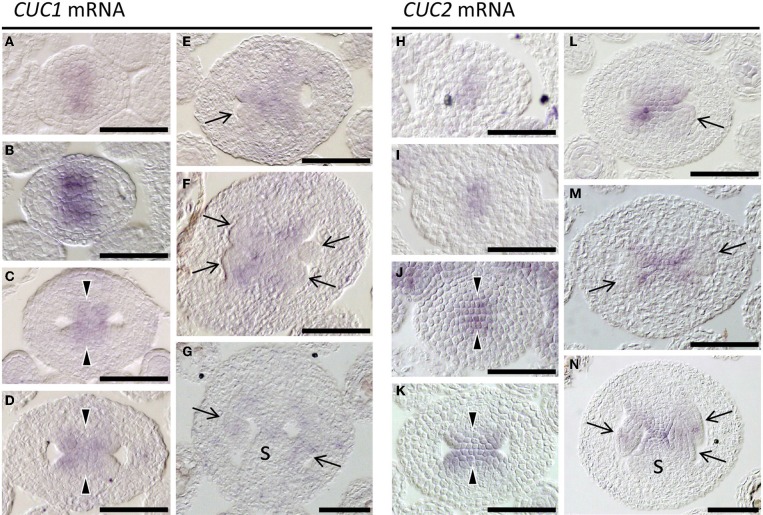
**Expression of *CUC1* and *CUC2***. *In situ* hybridization on transverse sections of developing wild type (L*er*) gynoecia regenerated from calli. Probed with *CUC1* **(A–G)** and *CUC2* **(H–N)**. Sections are prepared from gynoecia at stage 6 **(A,H)**, stage 7 **(B,I)**, stage 8 **(C,J)**, early stage 9 **(D,K)**, mid stage 9 **(E,L)**, late stage 9 **(F,M)**, and stage 10 **(G,N)**. Arrow, ovule; arrowhead, CMM; s, septum. Scale bars are 50 μm.

Expression patterns of *CUC1* and *CUC2* during gynoecium development have been reported only partially (Ishida et al., 2000; Takada et al., 2001; Nahar et al., 2012; Galbiati et al., 2013). We therefore carried out detailed expression analysis. Upon initiation of the gynoecial primordium, their expression was detected at its apical center, where the cleft of the future gynoecium cavity will form (Figures 3A,H). When the primordium became cylindrical, *CUC1* and *CUC2* expression was detected in the adaxial region of the medial wall, from which the CMM develops (Figures 3B,I). The area of *CUC1* expression domain was broader than that of *CUC2*. When the CMMs began to form, expression of both genes was detected throughout the bulge (Figures 3C,D,J,K). Their expression was missing in the developing ovule primordia but present in the remaining part of the CMMs (Figures 3E,L). Later, transcripts of *CUC1* and *CUC2* were both detected at the base of ovule primordia, the fused region of the septum, and in ovules (Figures 3F,G,M,N). These results are consistent with the role for *CUC1* and *CUC2* in CMM formation. Expression patterns from stage 7 to stage 10 are summarized in Supplementary Figure 1 and Supplementary Table 1.

### *CUC1* and *CUC2* are required for STM expression and prevent differentiation of CMM cells

**Figure 4:**
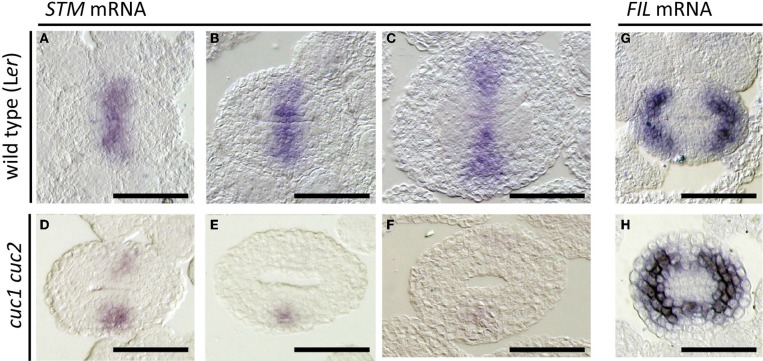
**Effect of *cuc1 cuc2* on *STM* and *FIL* expression**. *In situ* hybridization on transverse sections of developing wild type L*er* **(A–C,G)** and *cuc1 cuc2* **(D–F,H)** gynoecia regenerated from calli. Probed with *STM* **(A–F)** and *FIL* **(G,H)**. Sections are prepared from gynoecia at stage 8 **(A,D,G,H)**, early stage 9 **(B,E)** and mid stage 9 **(C,F)**. Scale bars are 50 μm.

The class I KNOX gene *STM* plays a critical role in maintaining shoot meristem activity and is also required for proper gynoecium development. As reported previously, *STM* expression was detected along the carpel margins of early gynoecia (Figure 4A; Long et al., 1996) and continued in the CMM while it was missing in ovule primordia (Figures 4B,C). In *cuc1 cuc2*, by contrast, *STM* expression was greatly reduced (Figures 4D–F). Notably, its expression tended to be absent on the adaxial side of the carpel margins while it remained on the abaxial side. These results show that *CUC1* and *CUC2* are required for *STM* expression in the CMM.

Expression of the *FIL* gene is detected in the future valve region while it is excluded from the carpel margins (Dinneny et al., 2005; Figure 4G). Together with its close homolog *YAB3*, it is required for valve development. In *cuc1 cuc2*, *FIL* expression extended toward the carpel margins and formed a continuous ring (Figure 4H). These results are consistent with the reduction of carpel margin structures in *cuc1 cuc2* and indicate that *CUC1* and *CUC2* prevent valve differentiation at the carpel margins.

### MicroRNA resistant versions of *CUC1* and *CUC2* genomic fragments cause expansion of the CMMs

**Figure 5:**
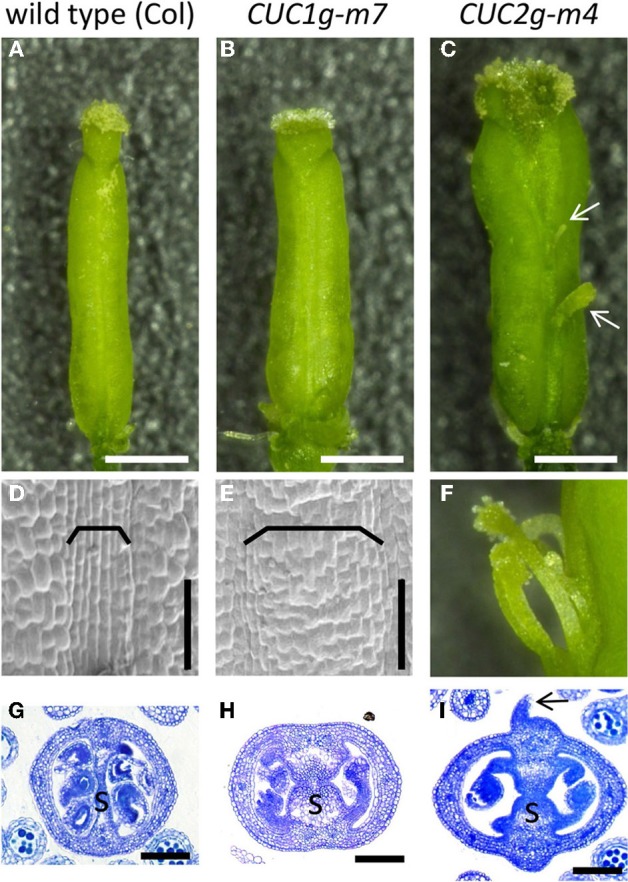
**Gynoecium phenotype of *CUC1g-m7* and *CUC2g-m4***. Mature gynoecia of wild type Col **(A,D,G)**, *CUC1g-m7* **(B,E,H)**, and *CUC2g-m4* **(C,F,I)**. Whole view **(A–C)**, scanning electron micrograph of replum **(D,E)**, close up view of filamentous structures **(F)** and transverse sections of ovary **(G–I)**. Arrows in **(C,I)** indicate filamentous structures. Brackets indicate repla. s, septum. Scale bars are: **(A–C)**, 500 μm; **(D,E,G–I)**, 100 μm.

We next examined the role of microRNA-dependent regulation of *CUC1* and *CUC2* in CMM formation. To this end, we used transgenic plants carrying genomic fragments of *CUC1* or *CUC2* that carry silent mutations in the target sequences of miR164 (*CUC1g-m7* and *CUC2g-m4*, respectively). These plants exhibited expansion of carpel margin structures including the replum (Figures 5A–F) and the septum (Figures 5G–I). In addition, the abaxial surface of carpel margins in *CUC2g-m4* was swollen and produced filamentous structures (Figures 5C,F,I; Nikovics et al., 2006).

**Figure 6:**
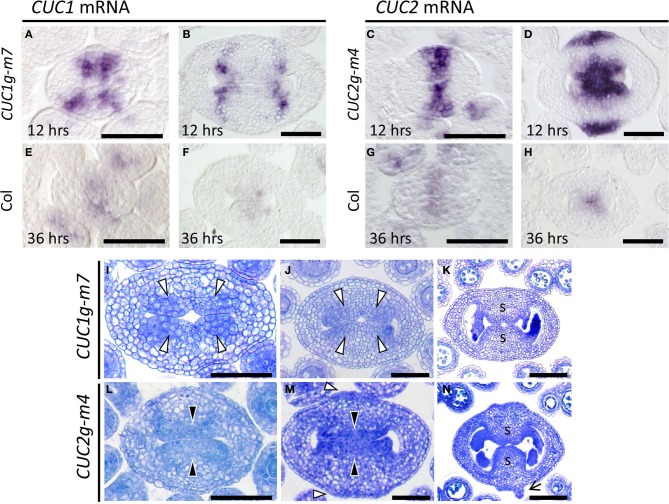
**CMM development in *CUC1g-m7* and *CUC2g-m4*. (A–H)** *In situ* hybridization on transverse sections from developing gynoecia of *CUC1g-m7* **(A,B)**, *CUC2g-m4* **(C,D)** and wild type Col **(E–H)** probed with *CUC1* **(A,B,E,F)** and *CUC2* **(C,D,G,H)**. Staining reaction was carried out for 12 h in *CUC1g-m7* **(A,B)** and *CUC2g-m4* **(C,D)**, and for 36 h in Col **(E–H)**. Sections are prepared from gynoecia at stage 6 **(A,C,E,G)** and late stage 9 **(B,D,F,H)**. **(I–N)** Transverse sections from developing gynoecia of *CUC1g-m7* **(I–K)** and *CUC2g-m4* **(L–N)** at early stage 9 **(I,L)**, late stage 9 **(J,M)**, and stage 11 **(K,N)**. Black arrowheads indicate CMMs at normal positions and white arrowheads indicate those at abnormal positions. Arrow in **(N)** indicates a primordium of a filamentous structure. s, septum. Scale bars are 50 μm.

Expression of *CUC1* and *CUC2* was examined to access the effect of the silent mutations introduced into the transgenes. In *CUC1g-m7*, *CUC1* mRNA initially accumulated in a broad region around the carpel margins with four peaks of staining (Figure 6A), which later dissolved into four discrete spots (Figure 6B). In *CUC2g-m4*, *CUC2* mRNA was first detected broadly throughout the carpel margins (Figure 6C) and later it split into adaxial and abaxial ends (Figure 6D). In both transgenic plants, stronger signals were observed in shorter staining time than in wild type (12 vs. 36 h), indicating that the levels of *CUC1* and *CUC2* mRNA was significantly elevated (compare Figures 6A–D with 6E–H).

We next examined early gynoecium development in these transgenic plants. In *CUC1g-m7*, CMMs were duplicated and initiated at four positions (Figure 6I, white arrowheads) that corresponded to the peaks of *CUC1* mRNA accumulation (Figure 6A). Each pair of the duplicated CMMs grew adaxially as a congenitally fused tissue and contacted each other at the center to undergo post-genital fusion, forming a thicker septum than that of the wild type (Figures 6J,K). The boundary of the fused CMMs was slightly depressed, forming a small central space after the fusion (Figure 6K). In *CUC2g-m4*, CMMs developed on the adaxial side and were broader compared to the wild type (Figure 6L). In addition, *CUC2g-m4* produced ectopic meristematic tissues as indicated by densely cytoplasmic cells on the abaxial side (Figure 6M, white arrowheads). These meristematic tissues further expanded and initiated primordia of filamentous structures on their flanks (Figure 6N, arrow). We interpret these meristematic tissues as ectopic CMMs, although they lack ability to form ovules. Together, the results show that disruption of miR164-mediated regulation of *CUC1* and *CUC2* strongly affected the size, positioning, and number of the CMMs.

**Figure 7:**
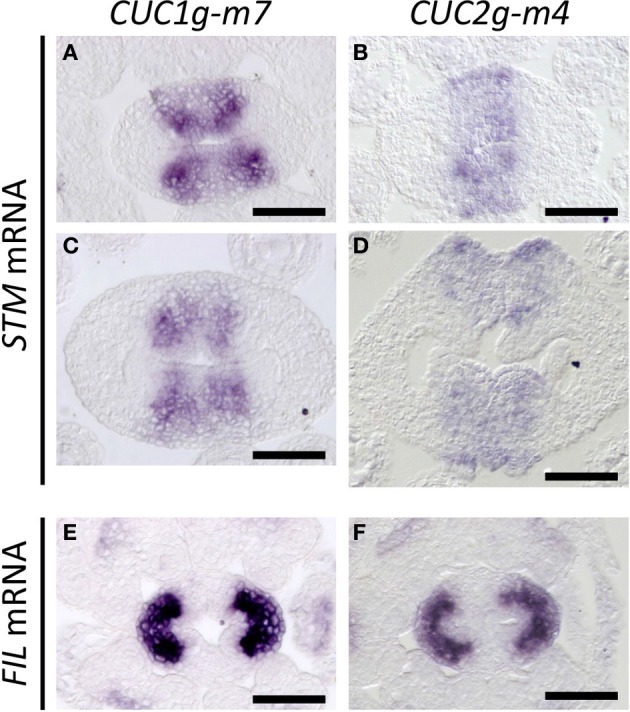
***STM* and *FIL* expression in *CUC1g-m7* and *CUC2g-m4***. *In situ* hybridization on transverse sections from developing gynoecia of *CUC1g-m7* **(A,C,E)** and *CUC2g-m4* **(B,D,F)** probed with *STM* **(A–D)** and with *FIL* **(E,F)** at early stage 9 **(A,B)**, late stage 9 **(C,D)** and stage 8 **(E,F)**. Scale bars are 50 μm.

In *CUC1g-m7*, expression of *STM* was laterally extended compared to that in wild type (compare Figures 7A with 4B), and four strong staining peaks were found within the expression domain, showing a strong correlation with the pattern of *CUC1* expression in this background (compare Figures 7C with 6B). Expression of *STM* was also broader in *CUC2g-m4* than in wild type and was detected throughout the carpel margins including outermost cells on the abaxial side, in which wild type did not accumulate *STM* transcripts (compare Figures 7B to 4B). This ectopic expression of *STM* continued in ectopic CMMs on the abaxial side (Figure 7D). On the other hand, *FIL* expression was not detected in the carpel margins of *CUC1g-m5* and *CUC2g-m7* as in the wild type (Figures 7E,F). These results indicate that elevated and ectopic levels of *CUC* gene expression in *CUC1g-m7* and *CUC2g-m4* cause increased meristematic activity of the CMMs.

## Discussion

Our results demonstrate that *CUC1* and *CUC2* play critical roles in formation and positioning of CMMs, which are central tissues for generating internal gynoecial organs. The loss of *CUC1* and *CUC2* activity caused severe reduction and altered positioning of CMMs, indicating that the previously reported defects in ovule and septum formation (Ishida et al., 2000; Galbiati et al., 2013) were due to the failure of forming these meristematic tissues. Moreover, *CUC1g-m7* and *CUC2g-m4* plants resulted in duplication and expansion of CMMs, and the positions of the duplicated CMMs in each transgenic plant are associated with the peaks of *CUC1* or *CUC2* transcripts, respectively. These results indicate that *CUC1* and *CUC2* promote CMM formation and, possively through the interaction with miR164, they are required for correct positioning of the CMMs.

Expression of *STM* was strictly dependent on *CUC1* and *CUC2* activities in the CMMs. This result is consistent with the previously reported function of *STM* in ovule formation (Scofield et al., 2007) and supports that *CUC1* and *CUC2* act upstream of *STM* in CMM formation. It has been suggested that *CUC1* and *CUC2* promote ovule development partly though activating a cytokinin pathway (Galbiati et al., 2013) and *STM* can promote cytokinin biosynthesis genes in seedling apices (Jasinski et al., 2005; Yanai et al., 2005). Our results are thus consistent with the idea that activation of *STM* expression by *CUC1* and *CUC2* promotes cytokinin production, which in turn contributes to ovule formation. Despite significant reduction of *STM* transcripts in *cuc1 cuc2* double mutant gynoecia, they do not show a split carpel phenotype, which has been reported for weak *stm* mutant alleles (Endrizzi et al., 1996) and inducible *STM* RNAi plants (Scofield et al., 2007). This aspect of carpel phenotype may reflect earlier function of *STM*, which is already expressed in the floral meristem before carpel initiation (Long et al., 1996). Alternatively, the remaining *STM* expression on the abaxial side of *cuc1 cuc2* carpel margins (Figures 4D–F) may be sufficient to prevent split of carpels.

In contrast to *STM*, the area of *FIL* expressing cells was greatly reduced in *cuc1 cuc2*, indicating that *CUC1* and *CUC2* negatively affect *FIL* expression. This result fits to the model in which factors responsible for carpel margin formation and those responsible for valve/valve margin formation counteract each other, as has been proposed based on interactions among *RPL*, *BP*, *FIL*, *JAG*, and *AS1/2* genes (Alonso-Cantabrana et al., 2007; Gonzalez-Reig et al., 2012). Because the STM protein has shown to physically interact with the carpel margin factor RPL (Byrne et al., 2003; Smith and Hake, 2003), it would be possible that the activation of *STM* expression by the *CUC* gene increases the amount of the STM/RPL complex in CMMs, thereby antagonizing the valve/valve margin factors including *FIL*.

Our results show that activation of the class I KNOX gene *STM* by *CUC1* and *CUC2*, a critical regulatory step during embryonic shoot meristem formation (Aida et al., 1999; Takada et al., 2001; Hibara et al., 2003), also occurs during CMM formation. The same regulatory relationship is also found in the formation of leaf margin structures (Kawamura et al., 2010) and is conserved among eudicots (Blein et al., 2008). Furthermore, the important roles for auxin and miR164 in regulating expression of *CUC* genes are also conserved among the processes of shoot meristem, leaf margin and carpel margin formation (Aida et al., 2002; Furutani et al., 2004; Nikovics et al., 2006; Larue et al., 2009; Koyama et al., 2010; Bilsborough et al., 2011; Galbiati et al., 2013). Further investigation on how these common regulatory factors are integrated into each developmental context and their possible relation with context-specific regulatory factors such as floral homeotic genes will be important to understand how unique shapes of different organs are formed.

## Author contributors

Yuri Kamiuchi, Masao Tasaka, and Mitsuhiro Aida designed the research. Yuri Kamiuchi, Kayo Yamamoto, Masahiko Furutani, and Mitsuhiro Aida performed the research. Yuri Kamiuchi and Mitsuhiro Aida analyzed the data and wrote the paper.

### Conflict of interest statement

The authors declare that the research was conducted in the absence of any commercial or financial relationships that could be construed as a potential conflict of interest.
