# Supplementary figures and images for: The CUC1 and CUC2 genes promote carpel margin meristem formation during Arabidopsis gynoecium development
Source: Front Plant Sci. 2014 Apr 30;5:165. doi: 10.3389/fpls.2014.00165 (PMC4012194; doi:10.3389/fpls.2014.00165)

Supplementary Figure 1. Schematic diagram of *CUC1* and *CUC2* expression.

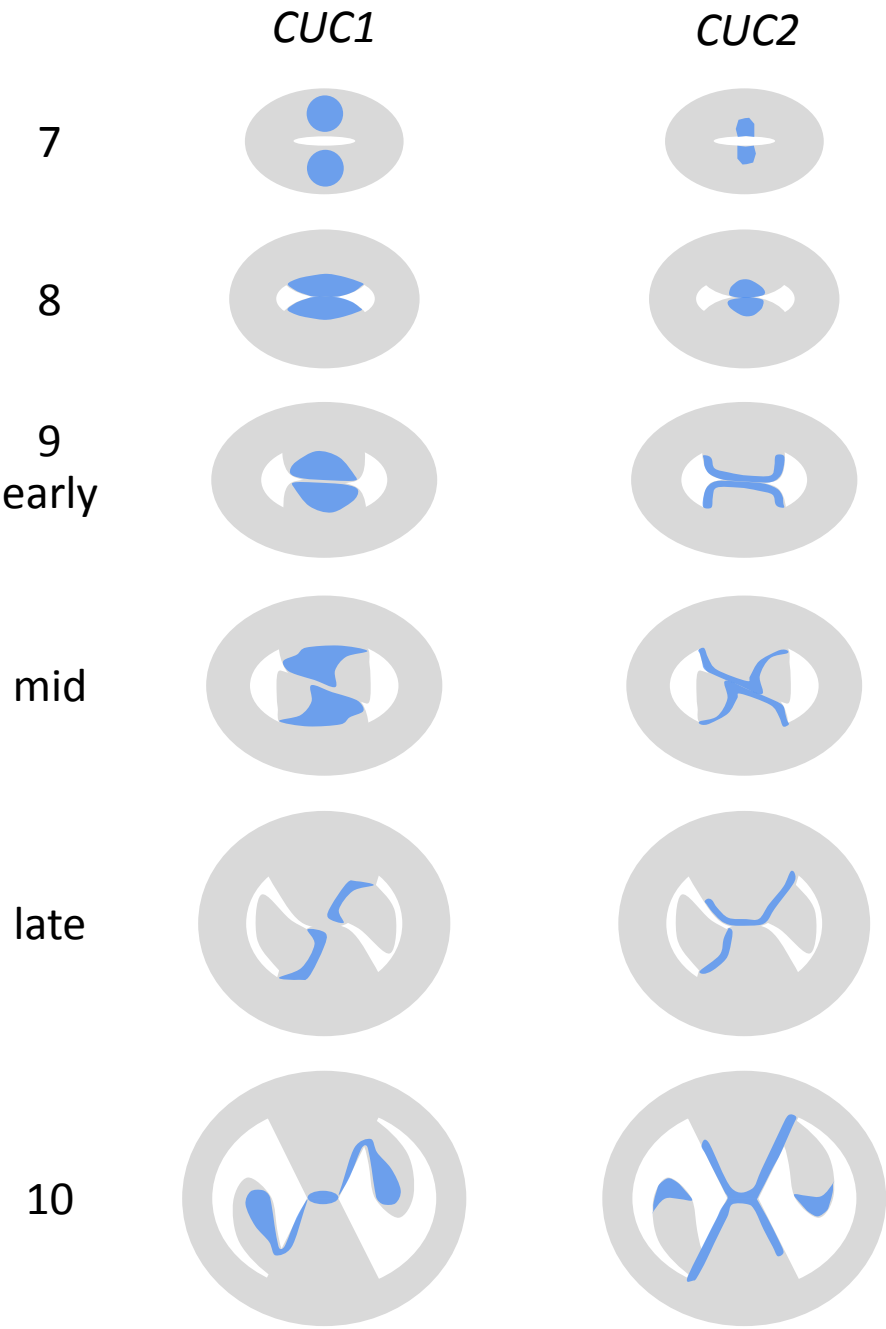

Supplement: Supplementary file 1 [file DataSheet1.PDF]
