# Supplementary material for: The CUC1 and CUC2 genes promote carpel margin meristem formation during Arabidopsis gynoecium development
Source: Front Plant Sci. 2014 Apr 30;5:165. doi: 10.3389/fpls.2014.00165 (PMC4012194; doi:10.3389/fpls.2014.00165)
Supplement: Supplementary file 2 [file DataSheet2.PDF]

Supplementary Table 1. Expression patterns of *CUC1* and *CUC2*

Stage*	*CUC1*	*CUC2*
7	Broadly along carpel margins	Strongly in the adaxial side of carpel margins
8	Weakly in the adaxial side of carpel margins	Strongly in the adaxial side of carpel margins
Early 9	Laterally in CMMs	Weakly in the whole CMMs
Mid 9	Weakly in the whole CMMs	Strongly along the boundary between CMM and ovule primordium
Late 9	Ovule primordium	Septum tips, ovule primordium
10	Weakly in the septum protoderm	Septum protoderm and ovule primordium

*Flower stage (Smith et al.,1990). Stage early 9, mid 9 and late 9 correspond to anther stage 5, 6 and 7, respectively (Sanders et al., 1999).

References

Sanders, P.M., Bui, A.Q., Weterings, K., Mcintire, K.N., Hsu, Y.C., Lee, P.Y., Truong, M.T., Beals, T.P., and Goldberg, R.B. (1999). Anther developmental defects in *Arabidopsis thaliana* male-sterile mutants. *Sex. Plant Rep.* 11**,** 297-322.

Smyth, D.R., Bowman, J.L., and Meyerowitz, E.M. (1990). Early flower development in *Arabidopsis*. *Plant Cell* 2**,** 755-767.
